# Latent profiles and influencing factors of quality of life among patients with colorectal cancer and an enterostomy in Southwest China: A multicenter cross-sectional study

**DOI:** 10.1016/j.apjon.2025.100745

**Published:** 2025-06-18

**Authors:** Xia Li, Xiaoyu Liu, Xiaolian Deng, Hua Zhang, Jiayi Su, Li Yuan, Aixin Zhou

**Affiliations:** a Department of Cardiothoracic Surgery, The First Affiliated Hospital of Chongqing Medical University, Chongqing, China; b Department of Nursing, The First Affiliated Hospital of Chongqing Medical University, Chongqing, China; c Gastrointestinal Cancer Center, Chongqing University Cancer Hospital & Chongqing Cancer Institute & Chongqing Cancer Hospital, Chongqing, China; d Gastrocolorectoanal Surgery, Daping Hospital, Third Military Medical University, Chongqing, China

**Keywords:** Colorectal cancer, Enterostomy, Quality of life, Latent profile analysis, Psychosocial factors, Nursing care

## Abstract

**Objective:**

This study aimed to identify latent profiles of quality of life (QoL) among patients with colorectal cancer and an enterostomy, and to explore factors associated with each profile.

**Methods:**

This multicenter cross-sectional study was conducted from August to November 2024 using convenience sampling. Patients diagnosed with colorectal cancer and living with a colostomy were recruited from gastrointestinal surgical wards and stoma outpatient clinics at three tertiary hospitals in Chongqing, China. Data were collected using the General Information Questionnaire, Stoma Quality of Life Scale, Medical Coping Modes Questionnaire, Social Isolation Scale for patients with colorectal cancer, and the Patient Health Questionnaire-9 (PHQ-9). Latent profile analysis (LPA) was employed to identify QoL subgroups, and multinomial logistic regression was used to examine influencing factors.

**Results:**

A total of 304 patients were included. Three distinct QoL profiles were identified: “Low Impact” (29.93%), “Moderate Equilibrium” (29.93%), and “High Social Support” (40.13%). Key influencing factors included self-care ability, postoperative duration, stoma type, stoma acceptance, coping style, level of social isolation, and depressive symptoms (*P* ​< ​0.05).

**Conclusions:**

Patients with colorectal cancer and an enterostomy exhibit diverse quality of life profiles following stoma surgery. Individualized nursing interventions tailored to profile characteristics may help improve their well-being and overall outcomes.

## Introduction

The latest global cancer statistics for 2022 reveal that there were approximately 1.92 million new cases of colorectal cancer, accounting for 9.6% of all cases, making it the third most common disease after lung and breast cancer. And there were 900,000 deaths due to this condition, representing 9.3% of all cancer-related deaths, second only to lung cancer,^1^ which poses a serious threat to human health. As the aging of population in China,^2^ the incidence of colorectal cancer is on the rise.^3^ Compared to the 700,000 patients with intestinal stoma in Europe,^4^ the number of such victims in China has surpassed one million, with around 100,000 new cases diagnosed each year.^5^ Enterostomy is a common surgical procedure performed for the treatment of colorectal cancer, where a portion of the intestinal tract is surgically brought through the abdominal wall to create an opening, allowing waste namely stool and mucus to be emptied and diverted outside the body, and it can be either temporary stoma or permanent stoma, depending on the condition and treatment strategies of patients.^6^^,^^7^ While patients can benefit a prolonged life after surgical stoma, they also have to bear unimaginable sufferings from both physical and psychological challenges and issues such as discomfort from stoma gas or noise, constipation, changes in sexual activities, unsightly body appearance, changes in clothing, as well as sense of social isolation or depression, all of which can severely affect the patient’s quality of life.^8^^,^^9^

Quality of life is an important indicator used to assess the recovery status and overall health level of patients with an enterostomy, covering multiple dimensions such as physiological function, psychological well-being, social adaptability, and the individual’s perception and response to the disease.^10^^,^^11^ Quality of life is directly relevant to the daily life experience of these patients, but it is also linked to their recovery process, treatment compliance, and the status of long-term survival outcomes. Patients with a poor quality of life typically face psychological challenges such as depression and anxiety, which can lead to a loss of confidence in their disease treatment and a lack of compliance with medical recommendations, ultimately affecting the prognosis of the disease.^12^ Thus, improving patients’ quality of life after stoma creation has become a key focus of clinical care and rehabilitation research in Chinese medical institutions. The quality of life of stoma patients in China is generally lower compared to some other countries, with many experiencing negative psychological states particularly anxiety. This is true for patients with temporary stomas, or those who have undergone surgery more recently, as they are prone to report an unsatisfactory quality of life.^13^ Importantly, researchers have identified additional factors that affect the quality of life of enterostomy individuals, including financial pressure, level of social support, stoma-related complications, disease awareness, and self-efficacy.^14^

Social isolation is used to describe individual behaviors and states of social avoidance, such as paucity of contacts with others, concealment of self-emotions, and specific negative emotional experiences (e.g., loneliness, isolation, and meaninglessness).^15^ Patients with an enterostomy who experience social isolation may be more prone to negative emotions like anxiety and depression, which can negatively impact their quality of life. Coping is a self-regulating process that involves behavioral and cognitive strategies, with common coping styles including confrontation, avoidance, and resignation, and negative coping styles having a significant adverse effect on the patient’s postoperative quality of life and psychological well-being.^16^ Previous studies have highlighted the impact of depression and social support on quality of life; however, there is limited research on the influence of social isolation and coping styles on the quality of life of patients with an enterostomy.

Current research on the quality of life of patients with an enterostomy in China primarily relies on variable-centered cross-sectional analyses, which focus on the predictive performance within a single dimension. Unfortunately, these approaches are limited in capturing the heterogeneity of patient populations, as different subgroups may present unique combinations of physiological, psychological, and social functions that require tailored intervention strategies. Although some scholars have advocated for the use of individual-centered analysis methods, relevant research within the enterostomy population is still in exploratory stages. Latent profile analysis, which is individual-centered and employs exogenous variables to determine potential characteristics,^17^ can reveal the heterogeneity within the patient group and provide a theoretical basis for personalized care. The results of the study are expected to optimize clinical intervention pathways, enhancing the overall effectiveness of care, and hold significant theoretical and practical value in improving the quality of life for patients with an enterostomy.

Taken together, the present study not only considers general demographic data but also includes depression, social support, social isolation, and coping styles as possible influencing factors. The aim is to explore latent categories of quality of life among intestinal stoma patients and analyze the influencing factors of these categories. The findings are expected to offer valuable insights for the development of personalized intervention programs and serve as a basis for clinical care and rehabilitation treatment.

## Methods

### Study participants

Between August and November 2024, patients with colorectal cancer who had undergone enterostomy were recruited from both inpatient gastrointestinal surgery departments and stoma outpatient clinics at three tertiary Grade A hospitals in Chongqing. Inclusion criteria were as follows: (1) diagnosis of colorectal cancer with initial enterostomy; (2) age ≥ 18 years; and (3) willingness to participate voluntarily. Exclusion criteria included: (1) presence of distant metastases or other malignant tumors; (2) cardiopulmonary failure or other severe physical conditions; and (3) cognitive impairment, disorientation, uncooperative behavior, or difficulty in verbal expression.

Sample size was calculated based on Kendall’s principle, which recommends 10 to 20 times the number of independent variables for multivariate analysis.^17^^,^^18^ With 25 independent variables and an estimated 20% attrition rate, a minimum of 300 participants was required. A total of 320 patients were initially enrolled. Thirteen patients declined to complete the questionnaire, and three were excluded due to incomplete or invalid responses. Consequently, data from 304 patients who had undergone enterostomy were included in the final analysis.

### Study tools

#### General Information Questionnaire

Through a comprehensive literature review and several discussions with the research team, a General Information Questionnaire was jointly designed with items including gender, age, marital status, education background, occupation, per capita monthly household income, night sleep duration, self-care status of stoma, self-acceptance of stoma, perceived disease degree, previous disease history, stoma type, surgical type, postoperative time, and complications etc., with 15 items in total.

#### Stoma Quality of Life Scale (Stoma-QOL)

The widely applied Stoma-QOL scale was developed by Prieto et al.^19^ for ostomy patients to measure their quality of life after stoma surgery, and was translated into Chinese and revised by Wu Xue et al.^20^ for a wider application. This instrument includes four dimensions with a total of 20 items. The dimension of social relations covers six items, the impact of ostomy bags on patients also has six items, the dimension of relations to family and close friends consists of five items, and the dimension of physical and mental conditions contains three. These items were designed with four-level Likert questions and the score is given from 1 to 4 for responses from “always” to “never”. The total score ranges from 20 to 80. Higher scores represent higher quality of life. The Cronbach’s α coefficient for this scale is 0.956 in this study.

#### Social Isolation Scale for patients with colorectal cancer

The measurement tool of social isolation is based on the research of Wang Wen et al.^21^ in 2022, and this scale is employed to assess the level of social isolation in colorectal patients. It contains two dimensions with 16 items. The social isolation includes four items and emotional isolation dimension consists of 12 items. A 5-point Likert scale is adopted, with scores ranging from 1 to 5, corresponding to responses from “very inconsistent” to “very consistent”, and the total score falls between 16 and 80 points. Higher scores indicate higher levels of social isolation. The content validity index at the item level of the scale was 0.86–1.00, and the content validity index at the scale level was 0.95. Exploratory factor analysis extracted two common factors, with a cumulative variance contribution rate of 70.17%. Confirmatory factor analysis showed a favorable goodness-of-fit. The Cronbach’s α coefficient of the overall scale was 0.923. The Cronbach’s α coefficient is 0.973 in the scale in this study.

#### Patient Health Questionnaire-9

The Patient Health Questionnaire-9 (PHQ-9)^22^ was developed following the nine diagnostic criteria outlined in the *Diagnostic and Statistical Manual of Mental Disorders* issued by the American Psychological Association. It is a tool consisting of nine items and applied to assess depression severity in patients. A 4-level Likert scale is used and the score is given from 0 to 3, corresponding to responses from “Not at all” to “Nearly every day”. The total score ranges from 0 to 27 and higher scores indicate more severe depression. The Cronbach’s α coefficient is 0.901 in this study scale.

#### Medical Coping Modes Questionnaire

This questionnaire was initially proposed by Feifel et al.^23^ in 1987 and subsequently translated into Chinese and revised by Shen Xiaohong et al.^24^ It is designed to measure the coping strategies of patients to deal with disease-related stress. It consists of three domains confrontation, avoidance, and acceptance-resignation, with 8 items in the confrontation domain, 7 items in the avoidance domain and 5 items in the acceptance-resignation. The scoring method adopts the 4-level Likert scale. Each item is assigned a score ranging form 1 to 4 points, from low to high, with a total score of 20–80 points. Reverse scoring is applied to eight items (No. 1, 4, 9, 10, 12, 13, 18, and 19). A higher average score for each item of the dimension indicates a greater tendency for the patient is to adopt that particular coping strategy. The Cronbach’s α coefficients of the three described domains are 0.896, 0.835, and 0.951, respectively.

### Data collection and quality control methods

The investigators are two designated postgraduate students from the research team, who have received standardized training prior to the survey. They are required to explain the purpose, significance and notes to fill the questionnaires to participants by using standardized guiding terms, which should be completed with the patient’s consent. Considering that most of the participants in this study were elderly patients with relatively limited comprehension ability and educational level to common medical knowledge, for those patients who were unable to complete the questionnaire on their own, the researchers offered assistance in patient explanation in a neutral tone. In addition, we helped some of them complete the questionnaire through a face-to-face question-and-answer approach. Each participant took approximately 15–20 minutes to complete the questionnaire. The questionnaires should be collected immediately upon completion and checked whether there are any missed answers. If so, the respondents are requested to refill the form in time. To improve the response rate, after the participants completed the questionnaires, we provided them with a nice gift (a storage bag for ostomy supplies) to express our gratitude for their cooperation. The results are independently handled by two team members and double checked in a timely manner, and those with regular answers, same options and duplicate cases are invalid questionnaires and should be removed from this survey.

### Statistical analysis

The Mplus 8.3 software was used to analyze the latent profiles of the quality of life of stoma patients. The quality of life scores of each item was applied as the exogenous variables, and 1 to 5 model analyses were carried out in turn. To better evaluate the accuracy of classifications, several model fit indicators^17^ were taken into account, mainly including Bayesian information criterion (BIC), adjusted Bayesian information criterion (aBIC), Akaike information criterion (AIC) and information entropy (Entropy). Lower values of the AIC, BIC, and aBIC represent higher effects of model fit; the Entropy value closer to 1 represents a more accurate classification. P values of both the Bootstrap Likelihood Ratio Test (BLRT) and the Lo-Mendell-Rubind (LMR) need to reach a significant level (*P* ​< ​0.05).

SPSS 26.0 statistical software was employed for data analyses. For measurement data that did not follow normal distribution, the results were presented as Median (p25, p75), while those conformed to normal distribution was expressed using mean ​± ​standard deviation (Mean ​± ​SD). Enumeration data were also addressed in terms of frequency and percentage. In a univariate analysis, the enumeration data were analyzed using either χ^2^ tests or Fisher’s exact tests. Meanwhile, *t*-tests or variance analyses were adopted for normally distributed measurement data, while the Kruskal–Wallis test was used for those did not normally distributed. Logistic regression was used to analyze the influencing factors of each potential category affecting quality of life, and test level α was set at 0.05.

## Results

### General information of patients with an enterostomy

A total of 307 questionnaires were handed out in this survey, yielding 304 valid responses, which corresponded to an effective response rate of 99.02%.

### Latent profile analysis results of social isolation in patients with an enterostomy

The findings of this survey revealed that 304 patients with an enterostomy had a quality of life score of 45.74 ​± ​12.13, which was at a medium level and this result is consistent with that of Zhou Jing et al.^13^ The patients’ quality of life scores were assessed using latent profile analysis, and models of potential categories from 1 to 5 were constructed based on the twenty items of the Stoma-QOL Scale, as shown in Table 1. As the number of categories increased, the values of AIC, BIC, and aBIC indicators gradually decreased. The third model presented a satisfactory entropy value, and both the LMRT and BLRT tests reached statistical significance. A comparison of the fitting effects between two adjacent models revealed that a *P* ​< ​0.05 value indicated that the k model was superior to the k-1 model.^2^ Taking both model indicators and application significance into consideration, the Model 3 was considered to be optimal fitting model for the quality of life of patients with an enterostomy for the study (Table 1).

**Table 1:** Model fitting Indicators of latent profiles for quality of life of patients with an enterostomy.

Model	AIC	BIC	aBIC	Entropy	LMR	BLRT	Category probability (%)
1C	14,772.540	14,921.221	14,794.361	–	–	–	–
2C	11,434.645	11,661.384	11,467.923	0.983	0.0000	0.0000	50.99/49.01
3C	10,771.972	11,076.768	10,816.705	0.975	0.0017	0.0000	29.94/29.93/40.13
4C	10,283.899	10,666.753	10,340.089	0.989	0.1500	0.0000	32.24/36.51/17.11/14.14
5C	9971.079	10,431.990	10,038.725	0.989	0.2563	0.0000	27.63/5.26/17.11/13.16/36.84

AIC, Akaike information criterion; BIC, Bayesian information criterion; aBIC, adjusted Bayesian information criterion; LMR, Lo-Mendell-Rubind; BLRT, Bootstrap Likelihood Ratio Test.

**Fig. 1:**
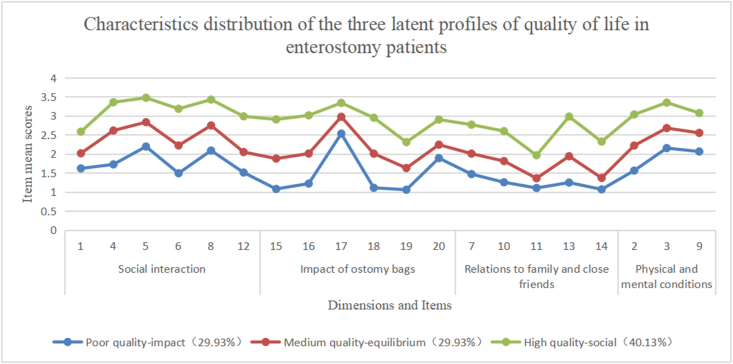
Characteristics distribution of the three latent profiles of the quality of life in patients with an enterostomy.

Thus a potential profile was plotted based on the quality of life categories, as shown in Fig. 1. Category 1 indicates “poor quality - impact” accounting for 29.93% (91/304); Category 2 indicates “medium quality - equilibrium” accounting for 29.93% (91/304), and Category 3 indicates “high quality - social” accounting for 40.13% (122/304).

### Univariate analysis of the latent profiles of quality of life in patients with an enterostomy

Univariate analyses results revealed statistically notable differences in indicators of self-care status of stoma, night sleep duration, acceptance of stoma, perceived disease degree, stoma type, postoperative time, complications, coping style, social isolation, and depression in the three groups (*P* ​< ​0.05, Table 2).

**Table 2:** Univariate analysis of general information and latent profiles of quality of life of participants.

Item	Category	Total cases (*n* ​= ​304)	Poor Quality - Impact (*n* ​= ​91)	Medium Quality - Equilibrium (*n* ​= ​91)	High Quality - Social (*n* ​= ​122)	Test statistic	*P* value
Sex [*n* (%)]	Male	191 (62.83)	55 (28.80)	60 (31.41)	76 (39.79)	0.613	0.736^a^
	Female	113 (37.17)	36 (31.86)	31 (27.43)	46 (40.71)		
Age [years, Median (p25, p75)]			61.00 (56.00, 71.00)	62.00 (56.00, 73.00)	61.00 (54.75, 71.00)	0.673	0.714
Marital status [*n* (%)]	Unmarried	3 (0.99)	2 (66.67)	1 (33.33)	0 (0.00)		0.258^b^
	Married	272 (89.47)	81 (29.78)	81 (29.78)	110 (40.44)		
	Divorced	8 (2.63)	1 (12.50)	1 (12.50)	6 (75.00)		
	Widowed	21 (6.91)	7 (33.33)	8 (38.10)	6 (28.57)		
Occupation [*n* (%)]	Retired	124 (40.79)	37 (29.84)	38 (30.65)	49 (39.52)	8.468	0.389^a^
	Employee	27 (8.88)	14 (51.85)	5 (18.52)	8 (29.63)		
	Individual	23 (7.57)	6 (26.09)	6 (26.09)	11 (47.83)		
	Farmer	95 (31.25)	23 (24.21)	31 (32.63)	41 (43.16)		
	Unemployed	35 (11.51)	11 (31.43)	11 (31.43)	13 (37.14)		
Education [*n* (%)]	Primary school and below	110 (36.18)	31 (28.18)	35 (31.82)	44 (40.00)		0.649^b^
	Junior high school	116 (38.16)	39 (33.62)	32 (27.59)	45 (38.79)		
	Senior high/polytechnic school	40 (13.16)	14 (35.00)	10 (25.00)	16 (40.00)		
	Vocational school	22 (7.24)	4 (18.18)	10 (45.45)	8 (36.36)		
	Bachelor or above	16 (5.26)	3 (18.75)	4 (25.00)	9 (56.25)		
Monthly household income per capita [*n* (%)]	< 2000	138 (45.39)	46 (33.33)	40 (28.99)	52 (37.68)	6.888	0.331^a^
	2000–4000	73 (24.01)	20 (27.40)	19 (26.03)	34 (46.58)		
	4000–6000	59 (19.41)	20 (33.90)	18 (30.51)	21 (35.40)		
	> 6000	34 (11.18)	5 (14.71)	14 (41.18)	15 (44.12)		
Stoma self-care status [*n* (%)]	Fully self-care	72 (23.68)	6 (8.33)	22 (30.56)	44 (61.11)	51.181	< 0.001^a^
	Partially	183 (60.20)	53 (28.96)	57 (31.15)	73 (39.89)		
	Not at all	49 (16.12)	32 (65.31)	12 (24.49)	5 (10.20)		
Night sleep duration [*n* (%)]	< 4 h	122 (40.13)	56 (45.90)	39 (31.97)	27 (22.13)	40.701	< 0.001^a^
	4–6 h	46 (15.13)	14 (30.43)	11 (23.91)	21 (45.65)		
	6–8 h	82 (26.97)	12 (14.63)	29 (35.37)	41 (50.00)		
	> 8 h	54 (17.76)	9 (16.67)	12 (22.22)	33 (61.11)		
Ostomy acceptance [*n* (%)]	Yes	172 (56.58)	16 (9.30)	43 (25.00)	113 (65.70)	124.068	< 0.001^a^
	No	132 (43.42)	75 (56.82)	48 (36.36)	9 (6.82)		
Perceived disease severity [*n* (%)]	Mild	130 (42.76)	9 (6.92)	30 (23.08)	91 (70.00)	112.283	< 0.001^a^
	Moderate	52 (17.11)	13 (25.00)	21 (40.38)	18 (34.62)		
	Severe	122 (40.13)	69 (56.56)	40 (32.79)	13 (10.66)		
Previous medical history [*n* (%)]	None	212 (69.74)	62 (29.25)	61 (28.77)	89 (41.98)	1.023	0.599^a^
	Yes	92 (30.26)	29 (31.52)	30 (32.61)	33 (35.87)		
Stoma type [*n* (%)]	Temporary	178 (58.55)	73 (41.01)	51 (28.65)	54 (30.34)	28.106	< 0.001^a^
	Permanent	126 (41.45)	18 (14.29)	40 (31.75)	68 (53.97)		
Surgical type [*n* (%)]	Ileostomy	135 (44.41)	46 (34.07)	43 (31.85)	46 (34.07)	3.909	0.142^a^
	Colostomy	169 (55.59)	45 (26.63)	48 (28.40)	76 (44.97)		
Post-operative time [*n* (%)]	≤ 1 month	49 (16.12)	27 (55.10)	10 (20.41)	12 (24.49)	44.099	< 0.001^a^
	1–3 months	53 (17.43)	23 (43.40)	13 (24.53)	17 (32.08)		
	3–6 months	62 (20.39)	15 (24.19)	21 (33.87)	26 (41.94)		
	6–12 months	47 (15.46)	13 (27.66)	22 (46.81)	12 (25.53)		
	> 1 year	93 (30.59)	13 (13.98)	25 (26.88)	55 (59.14)		
Complications [*n* (%)]	None	221 (72.70)	55 (24.89)	67 (30.32)	99 (44.80)	11.317	0.003^a^
	Yes	83 (27.30)	36 (43.37)	24 (28.92)	23 (27.71)		
Coping style [*n* (%)]	Confrontation	146 (48.03)	7 (4.79)	27 (18.49)	112 (76.71)	173.508	< 0.001^a^
	Avoidance	46 (15.13)	17 (36.96)	21 (45.65)	8 (17.39)		
	Resignation	112 (36.84)	67 (59.82)	43 (38.39)	2 (1.79)		
Social isolation [score, Median (p25, p75)]			66.00 (60.00, 71.00)	56.00 (44.00, 62.00)	32.00 (29.00, 36.00)	208.021	< 0.001^c^
Depression [score, Median (p25, p75)]			8.00 (5.00, 13.00)	3.00 (1.00, 6.00)	0 (0.00, 0.00)	176.655	< 0.001^c^

a Chi-square test.

b Fisher’s exact test.

c Kruskal–Wallis test.

### Multivariate analysis of the latent profiles of quality of life in patients with an enterostomy

Multivariate Logistic regression analysis was performed using three latent profiles of quality of life in patients with an enterostomy as dependent variables and the factors with statistical significance through univariate analysis as independent variables. Depression and social isolation scores were brought in with the measured values, and the remaining eight variables were assigned (Table 3). Multivariate Logistic regression analyses indicated that patients with higher scores of social isolation and depression and temporary stomas were associated with a greater likelihood of the “poor quality-impact”, as compared to the profiles of “medium quality-equilibrium” and “high quality-social”. Meanwhile, compared to the “poor quality-impact” and “high quality-social”, patients with a postoperative duration of 6–12 months and good self-care status of stoma were more likely to be classified as the “medium quality-equilibrium” type. Additionally, compared to the “poor quality-impact” and “medium quality-equilibrium”, patients with a postoperative duration of 3–6 months and a coping style confrontation had a higher probability of as “high quality-social” type (Table 4).

**Table 3:** Independent variable assignment.

Variables	Assignment method
Stoma self-care status	Completely ​= ​1, partially ​= ​2, not at all ​= ​3
Night sleep duration	< 4 h ​= ​1, 4–6 h ​= ​2, 6–8 h ​= ​3, > 8 h ​= ​4
Stoma acceptance	Yes ​= ​1, No ​= ​2
Perceived disease severity	Mild ​= ​1, moderate ​= ​2, severe ​= ​3
Stoma type	Temporary (Z_1_ ​= ​1, Z_2_ ​= ​0), permanent (Z_1_ ​= ​0, Z_2_ ​= ​1)
Post-operative time	≤ 1 month ​= ​1, 1–3 months ​= ​2, 3–6 months ​= ​3, 6–12 months ​= ​4, > 1 year ​= ​5
Complications	None ​= ​1, Yes ​= ​2
Coping style	Confrontation (Z_1_ ​= ​1, Z_2_ ​= ​0), avoidance (Z_1_ ​= ​0, Z_2_ ​= ​1), resignation (Z_1_ ​= ​0, Z_2_ ​= ​0)

**Table 4:** Multivariate Logistic regression results of latent profiles of quality of life in patients with enterostomy.

Item	*β* value	Standard error	Wald c^2^	*P* value	*OR* value	*95% CI*	
						Lower limit	Upper limit
**C3 vs. C1**^a^							
Postoperative time (3–6 months)	−2.988	1.183	6.383	0.012	0.050	0.005	0.512
Coping mode (confrontation)	−4.137	1.329	9.689	0.002	0.016	0.001	0.216
Social isolation score	0.220	0.048	20.552	< 0.001	1.246	1.133	1.370
Depression score	0.696	0.223	9.750	0.002	2.005	1.296	3.103
**C3 vs. C2**^a^							
Stoma acceptance (yes)	−1.530	0.700	4.780	0.029	0.216	0.055	0.854
Coping modes (confrontation)	−3.541	1.150	9.478	0.002	0.029	0.003	0.276
Social isolation	0.131	0.037	12.389	< 0.001	1.139	1.060	1.225
Depression	0.536	0.214	6.251	0.012	1.710	1.123	2.603
**C1 vs. C2**^b^							
Stoma type (temporary)	−1.455	0.547	7.071	0.008	0.233	0.080	0.682
Postoperative time (3–6 months)	1.653	0.773	4.576	0.032	5.220	1.149	23.728
Postoperative time (6–12 months)	1.501	0.760	3.903	0.048	4.485	1.012	19.878
Stoma self-care status (Completely)	1.648	0.772	4.561	0.033	5.199	1.145	23.599
Social isolation score	−0.089	0.033	7.461	0.006	0.915	0.858	0.975
Depression score	−0.159	0.067	5.675	0.017	0.853	0.748	0.972

C1, poor quality-impact type; C2, medium quality-equilibrium type; C3, high quality-social type; OR, odds ratio; CI, confidence interval.

a C3 reference group.

b C1 reference group.

## Discussion

### Characteristics of different latent profiles among patients with an enterostomy

The study findings indicated evident heterogeneity in patients’ quality of life follow stoma creation, which were divided into three latent profiles. The proportion of “poor quality-impact” accounted for 29.93%. This type of patients with an enterostomy had relatively lower scores in the two dimensions “relations with family and close friends” and “impact of ostomy bags on patients”. It might be related to the patient’s shame about their own ostomy, resulting in unwillingness to make too much physical contact with close friends and family, as well as the unpleasant smell, exhaust sound and excretion leakage of the ostomy bag. However, the item number 17 “My stoma makes me unattractive to the opposite sex” had a higher score, the reason for which might be the mean age of participants in this study was 61 (56.00, 71.75) years old, and older people pay less attention to their physical attractiveness than younger people. The proportion of “medium quality-equilibrium type” was 29.93%. Patients with an enterostomy of this type scored at a moderate level for the four dimensions of quality of life, while they had higher scores in the dimensions of “social interaction” and “physical and mental conditions”. It might be that although the ostomy bags and the relations to family and close friends had a greater impact on the patients.

Meanwhile, the patients were willing to socialize, and their physical and mental conditions are good, which reduced the impact of the ostomies on the quality of life. “High quality - social” accounted for 40.13%. The patients of this type had high scores in all dimensions, with good social skills, positive and optimistic attitudes, which might contributed to improving their quality of life. But items number 11 “I avoid close physical contact with friends” and number 19 “I worry my ostomy bag will come loose” had lower scores. It might be related to the insufficient self-management ability of the ostomy bag and the impact of ostomy on sexual activities. It has been reported that^25^ patients with an enterostomy in China have a low quality of sexual life, which is affected by several factors such as marital relationship, postoperative time, surgical type, as well as stoma type.

This study indicated that patients with a higher level of social isolation tend to be classified as “poor quality-impact”. Social isolation is used to describe individual behaviors and states in which an individual is likely to show social avoidance (e.g., avoidance of contact with family and close friends, concealment of self-emotions) and present specific negative emotional experiences (e.g., loneliness, isolation, meaninglessness).^15^ Patients with intestinal stomas are tend to be socially alienated and not willing to go out home due to leakage of ostomy bags, unsightly body image, etc. Their social activities are notably reduced, and they even have negative feelings like anxiety and self-stigmatisation, which greatly influence the quality of life. Wang et al.^26^ have reported that social isolation directly reduces the quality of life in elderly individuals (*β* ​= ​0.306), and depression (*β* ​= ​0.334) plays an evident role in mediating between social isolation and quality of life. However, the present study has suggested that depression can directly affect the quality of life of stoma patients, which is consistent with the findings of Siddiqui et al.^12^ reported depression and quality of life among patients with colorectal cancer. Depression can also trigger a number of physical health problems such as sleep disorders, loss of appetite or overeating, and serious psychological problems such as feeling low, helpless, and despair, greatly affecting the quality of life.^27^ The incidence of depression in patients with an enterostomy has been found as high as 41.6% based on the research of Tang et al.^7^ Health care professionals are suggested to pay more attention to the social isolation and depression of intestinal stoma patients, monitoring their status dynamically. Cognitive behavioral therapy,^28^ mindfulness therapy^29^ and positive psychological intervention^30^ are adopted to prevent and alleviate social isolation and depression in patients with an enterostomy.

In addition, this study demonstrated that patients with temporary stoma were more likely to be classified as “poor quality-impact”, which is consistent with the results of Zhou Jing et al.^16^ Temporary ostomies are mostly reversed after 3–6 months. Patients with temporary ostomies have a relatively short duration of ostomy adaptation and may lack sufficient self-management skills and psychological adjustment during this period. Besides, temporary ostomies are often ileostomies, which are characterized by high volume, thin, and irregular excretion of waste. This makes home management more challenging for patients and their families, potentially leading to complications such as irritant dermatitis, which significantly impacts the quality of life.^31^ Therefore, more attention is needed by temporary stoma patients, whose diets and self-management skills at home are to be properly directed, to minimize complications and improve quality of life.

This study further revealed that patients with an enterostomy 6–12 months after surgery had a higher probability of being classified as “medium quality-equilibrium”. Six to 12 months after the ostomy, the intestinal functions of patients gradually stabilized, the acceptance of the ostomy and proficiency of self-care skills improved, diet and exercise gradually recovered, and the quality of life was improved. Studies have shown^13^ that longer duration of ostomy use improves the quality of life. Therefore, medical professionals should prioritize patients with a short duration post-ostomy surgery, providing them more remote home management guidance. This includes monitoring the ostomy and surrounding skin, proper use of ostomy bags, psychological counseling, and social support, to help patients adapt more quickly to the ostomy and facilitate a higher quality of life. This study also showed that patients with good skills of stoma self-care were more likely to be classified as “medium quality-equilibrium type”. Stoma self-care abilities cover the daily self-health monitoring, stoma maintenance and management of patients. These are essential foundations for the long-term management of chronic diseases. Improving patients’ self-care abilities can reduce the consumption of medical resources while enhancing health-promoting behaviors, disease prevention and chronic disease management.^6^ Mastering full skills of stoma self-care can increase the self-identity and self-esteem of stoma patients, alleviate psychological stress, and contribute to better physical function recovery, often approaching the pre-disease state. Additionally, it is conducive to reducing the burden on family caregivers, enhancing social participation, and improving overall quality of life. For patients with an enterostomy who cannot perform good self-care skills, medical staff can direct them through stoma clinic follow-up, remote video guidance and organize networking activities to improve stoma self-care abilities, thereby reducing the burden on family caregivers as well as improving quality of life.

Patients with intestinal stoma 3–6 months after surgery were more likely to be classified as “high-quality-social”. Compared with those between 6 and 12 months after surgery, the quality of life was slightly improved, which is different from the results by Zhou Jing et al.^13^ It is likely that early complications related to ostomy are largely resolved or controlled 3–6 months after surgery, with the physical condition remaining relatively stable, while patients 6–12 months after surgery may experience complications at intermediate or late stages, such as stenosis or prolapse, which can negatively affect the patients' quality of life. Importantly, the period of 3–6 months post-surgery is a critical phase for the physical recovery of ostomy patients. During this time, sufficient family support and care are of vital significance for the rapid physical recovery, notable improvement in stoma self-care abilities of patients with good physical and mental health. However, between 6 and 12 months post-surgery, as time progresses, fatigue from home management may increase, and both family attention and social support may decline, which could negatively affect the patient’s psychological well-being and quality of life. The results of this study showed that there was a high probability that patients employed a confrontation coping style can be classified as “high quality-social”. Patients who held a positive attitude to the current situation of their own disease and stoma status can actively learn skills of stoma self-management, which contributes a lot to both physical and mental recovery, enhancing psychological resilience and self-efficacy, thereby facilitating a harmonious family and social relations as well as good quality of life.^32^ Therefore, medical staff should take measures to help patients with an enterostomy actively engage in their conditions, such as offering stomas teaching sessions, providing informative videos, demonstrating operation techniques of stoma self-care, and positive psychological support, all aimed at improving the patients' quality of life.

### Implications for nursing practice and research

First, for patients with low quality of life, it is important to prioritize screening for negative emotions such as social isolation and depression, then we should address these emotions through interventions namely cognitive-behavioral therapy, mindfulness and peer support to prevent further negative impacts on their quality of life. Second, patients with temporary stomas in a short time, may struggle to adapt to its presence. Meanwhile, thin stoma excretions can make self-care more challenging. Therefore, more attention should be given to those temporary stoma patients by providing training on home self-management through stoma workshops, boosting their confidence through peer education, and establishing a “stoma clinic-community-family” support system to enhance their self-care skills and help them adjust to the stoma more quickly. Third, the 3–6 month period after surgery is critical for patients with an enterostomy to handle changes in quality of life. Strong home guidance available within the first three months can notably improve their quality of life afterward. However, patients in subsequent 6–12 months require special attention, which differs from previous studies. Between 6 and 12 months, patients may experience a decline in quality of life again due to fatigue, reduced family attention, and mid-late complications. To address this, we should encourage family members and health care providers to maintaining onging support for patients' home management, offer psychological care, and encourage patients to actively face and accept their stomas to improve quality of life. Finally, future research should clarify the causal relationships between social isolation, depression, and quality of life. It should track the dynamic changes in quality of life across different patient profiles over time and develop stepped intervention programs to further improve patients' quality of life.

### Limitations

Despite the valuable contributions of this study, several limitations should be acknowledged. First, as a cross-sectional study, it could not establish causal relationships between the identified factors and the latent profiles of quality of life in patients with an enterostomy. Consequently, temporal sequencing and cause–effect associations remain undetermined. For instance, although several demographic and clinical variables were identified to be associated with different quality-of-life profiles, it remains uncertain whether these variables directly influence the observed quality-of-life patterns or are merely correlated with them.

Second, this study was limited to Chongqing, the largest city in Southwest China. Although the multi-center design enhanced the sample diversity in this region, we took study feasibility into consideration and convenience sampling was adopted. Even though three of the most representative hospitals were selected, they cannot fully reflect all the characteristics of patients with an enterostomy in Southwest China, and the research findings may not be fully generalizable to patients with an enterostomy nationwide. Geographical, cultural, and health care - resource disparities exist between different regions in China, which could potentially influence patient’s quality of life after enterostomy in ways. Future research should conduct stratified sampling in secondary hospitals and from other cities of Southwest China to verify the results.

Third, the study collected data using self-reported questionnaires, which might lead to potential recall bias and social desirability bias. Patients might not accurately recall certain experiences related to their stoma care or might present responses that they perceive to be more socially acceptable, potentially affecting the accuracy of data.

Finally, this study did not address potential changes in patients' quality of life over time due to disease progression, recurrence, or long-term adaptation to the stoma. Longitudinal studies are necessary for further understanding of the dynamic nature of quality of life in patients with an enterostomy and to develop more effective, time-sensitive interventions.

## Conclusions

This study classified the quality of life of intestinal stoma patients into three potential categories using latent profile analyses. Patients with higher levels of social isolation and depression with temporary stoma, were more likely to be classified as “low quality-impact” group. Patients with 6–12 months postoperative and full skills of stoma self-care belonged to the “medium quality-equilibrium” group. Patients with 3–6 months postoperative and a confrontation coping style were classified as “high quality-social” group. It is recommended that in clinical practice, medical care providers tailor personalized intervention programs according to the characteristics of different latent categories of stoma patients to promote their mental health and improve their quality of life. This study only recruited ostomy patients following colorectal surgeries from three tertiary grade A hospitals in Chongqing China as the research subjects. Future research could involve multi-center, large-scale samples to further analyze the characteristics of various latent profile categories of the quality of life for stoma individuals.

## Credit authorship contribution statement

**Xia Li:** Conceptualization, Data Curation, Formal Analysis, Investigation, Methodology, Software, Visualization, Writing - Original Draft, Writing - Review & Editing. **Xiaoyu Liu:** Methodology, Supervision, Project administration. **Xiaolian Deng:** Methodology, Supervision, Project administration. **Hua Zhang:** Conceptualization, Funding Acquisition, Resources, Supervision, Project administration, Writing - Review & Editing. **Jiayi Su:** Data Curation, Investigation. **Li Yuan:** Data Curation, Investigation. **Aixin Zhou:** Data Curation, Investigation. All the authors have read and approved the final manuscript.

## Ethics statement

All procedures involving human participants were conducted in accordance with the ethical standards of the institutional and/or national research committees, and with the 1964 Declaration of Helsinki and its later amendments or comparable ethical standards. Ethical approval for this study was obtained from the Ethics Committees of the following institutions: The First Affiliated Hospital of Chongqing Medical University (Scientific Research Review, Approval No. 2024-133-01), Daping Hospital, Third Military Medical University (Medical Research and Clinical Review, Approval No. 306, 2024), and Chongqing University Cancer Hospital (Approval No. CZLS2024220-A). All participants provided written informed consent.

## Data availability statement

The data that support the findings of this study are available from the corresponding author, HZ, upon reasonable request.

## Declaration of generative AI and AI-assisted technologies in the writing process

No AI tools/services were used during the preparation of this work.

## Funding

This study was supported by Chongqing Key Specialty Construction *Journal of Clinical Nursing* Quality Construction Project (Grant No. 0203 2023 No. 47202336); Project of Science & Technology Department of Sichuan Province (Grant No. 2022NSFSC0648). The funders had no role in considering the study design or in the collection, analysis, interpretation of data, writing of the report, or decision to submit the article for publication.

## Declaration of competing interest

The authors declared no conflict of interest.
